# Our Book Table

**Published:** 1884-05

**Authors:** 


### OUR BOOK TABLE.

SHAKESPEARE AS A PHYSICIAN: *Comprising every word which in any way relates to Medicine, Surgery or Obstetrics, found in the Complete Works of that Writer, with Criticisms and Comparisons of the same with the Medical Thoughts of To-day:* by J. PORTMAN CHESNEY, M. D. St. Louis: J. H. Chambers & Co., Publishers. 1884. Octavo, pp. 226.

Shakespeare has often been spoken of as "the myriad-minded bard." Members of all the liberal professions find in his works indications that this wonderful man possessed a knowledge, more or less complete, of intricate professional matters, that none but he who had made a special study of, could possess. But his plays are especially rich in medical references, and show that he must have been an attentive student of medical lore. Nor were his the mere generalizations of one of the dilettanti. In all the various departments of medicine he seemed to be fully abreast of the professional knowledge of the time in which he lived. How extensive and how technical some of this was, the ordinary reader of his works does not readily appreciate. It is only when the whole is collated, and the extracts intelligently compared, as in the book under notice, that the professional mind becomes fully impressed with his real greatness. Every student of the great poet, especially every medical man, would do well to examine this work, for it is an epitome of the medical knowledge of the time.

VULCANITE AND CELLULOID: *Instruction in Their Practical Working for Dental Purposes*: by S. ELDRED GILBERT, D. D. S. Philadelphia: The S. S. White Dental Manufacturing Co. 1884.

This is a manual of plain directions in the manipulation of the two bases for artificial teeth, now most extensively used. All the most approved methods, from the taking of the impression to the insertion of the finished piece, are detailed. There is no attempt to pad the book into the proportions of a large volume by the introduction of fanciful methods, or by intricate theoretical speculations concerning the philosophy of each step, but all is plain, practical, and concise. For the purpose for which it is intended, the instruction of students and young practioners, we know of nothing better.

THE TEETH, THEIR FORMATION, DISEASES AND TREATMENT: *A Popular and Scientific Guide for the General Public*; by THOMAS GADDES, L. D. S., ENG. AND EDIN. London : David Bogue, Publisher. Illustrated.

Any work from the pen of the accomplished editor of "The Dental Record" will be read with interest by every intelligent dentist. The manual under notice was not specially written for professional eyes, yet there is not a professional man who might not study it with profit, while for the layman it contains a mass of information that should enable him to thoroughly comprehend the growth and ordinary diseases of the dental organs. If every physician would master its contents, we should not so often observe the indications of ignorance of these organs that are exhibited by men from whom one might expect better things.

MINUTES OF THE NINETEENTH ANNUAL MEETING OF THE ILLINOIS STATE DENTAL SOCIETY, 1883.

From the late Secretary, but present President of this Society, Dr. Edmund Noyes, we have received the above very handsome volume.

The Illinois State Dental Society is largely made up of men who are widely known in the profession, and the annual report of their proceedings is looked for with constantly increasing interest. The minutes of the meeting for 1883 are in no respect inferior to those of any previous year. In some respects the volume is a great improvement upon its predecessors. The papers are excellent, and some of the illustrations are exquisite. Such a volume can only be the result of great labor and thorough fitness for the onerous task of the committee on publication.

THE NEW YORK MEDICAL JOURNAL.—The publication of the monthly journal known as the "Proceedings of the Medical Society of Kings" has been discontinued. Henceforth the transactions of that Society will be regularly published in *The New York Medical Journal,* which, by the way, is the very best medical weekly published in New York. Any of our readers who desire to remain thoroughly conversant with all branches of medical science, and who wish a journal that is at all times entirely reliable and trustworthy, should send in their names to D. Appleton & Co., its publishers. There is not a practicing dentist who would not be directly benefited to the extent of five times its subscription price, if he would attentively read it.

TRANSACTIONS OF THE AMERICAN DENTAL ASSOCIATION. *Twenty-third Annual Session, held at Niagara Falls, Aug.,* 1883. Philadelphia: The S. S. White Dental Manufacturing. Co.

This volume is extremely late in making its appearance, and as a whole it is of much less value than most of its predecessors. Only four formal papers were presented, and of these but two were of any special interest, while the discussions at no time were worthy the Society or the occasion. The representative American Dental Society must do better than this if it is to retain a respectable position. The publishers have done their work well, and it is a pity that they had not better materials at their command.

ZAHNARZTLICHER ALMANACH, *for the years* 1877-'78-'79-'80-'81. Edited by ADOLPH PETERMANN, D. D. S., Royal Prussian Approbated Dentist; Dentist to the Royal Family of Hohenzollern; Honorary member of the Free German Hochstifts; Honorary member of the American Dental Association, etc., etc.

This is an alphabetical list of the names of approbated dentists practicing in the German Empire, and in Austria, Hungary, etc., with the names of acknowledged dental societies, journals, etc., revised from year to year. A very convenient and valuable hand-book of reference.

NOTES ON THE OPIUM HABIT; by ASA P. MEYLERT, M. D. New York: G. P. Putnam's Sons.

It is a question whether the abuse of opium be not a greater evil than that of alcohol. At any rate, it is the duty of the physician to thoroughly comprehend the subject, and a perusal of this excellent address will do much toward that end.

THE ATLANTA MEDICAL AND SURGICAL JOURNAL.—This old southern favorite commences a new series with its March number, and in its new dress and cover presents a very handsome appearance. It will scarcely change the verdict of the world, however, when it again claims for Dr. Crawford W. Long, of Georgia, whose portrait it places prominently upon its title page, the original discovery of Anæsthesia. That question was long ago settled to the satisfaction of most people, and the credit given where it belongs, to a dentist. Even the ability of Dr. Sims, whose sympathy not unnaturally was enlisted through a pardonable sectional pride, was not sufficient to obtain a reversal of the decision.

The following books and pamphlets have also been received:

*A Memorial Meeting Relative to the Death of* THOS. L. BUCKINGHAM; under the auspices of the Dental Societies of Philadelphia, with an excellent portrait of the lamented Professor.

*Iodoform in Dental Surgery.* By C. F. W. Bodecker, D. D. S. New York.

*Closure of the Jaws* and *Removal of a Tumor.* By J. J. R. Patrick, Belleville, Ill.

*A Consideration of Temperament in Relation to the Teeth.* By James W. White, M. D., D. D. S., Philadelphia.

*The Bacteria.* By Charles S. Boynton, M. D., Brandon, Vermont.

*Decay and Preservation of the Teeth.* By Dr. J. E. Low, Chicago, Illinois.

*Removal of the Ovaries and Fallopian Tubes for Chronic Ovaritis, Ovarian Dysmenorrhœa, Neuralgia, etc.* By Thomas H. Hawkins, M. D., Denver, Col.

*Hysterical Convulsions (Reflex); Perineorrhaphy and Trachleorrhaphy.* By Thomas H. Hawkins, M. D., Denver, Col.

*Peroxide of Hydrogen in Suppurative Conjunctivitis and Mastoid Abscesses.* By A. E. Prince, M. D., Jacksonville, Ill.

*Aneurism of the Femoral Artery,* and *A Knife Wound of the Intestines.* By W. O. Roberts, M. D., Louisville, Ky.

*Verein der Zahnarzte in Rheinland und Westfalen. Sitzungen am* 14 *und* 15 *Oct.*, 1883, *zu Coln.*

